# A Study on the Harmonic Distortion of Seismic-Grade Sigma-Delta MEMS Accelerometers Using a Multiple Degree-of-Freedom Model

**DOI:** 10.3390/s23198222

**Published:** 2023-10-02

**Authors:** Xuefeng Wang, Penghao Zhang, Shijin Ding

**Affiliations:** State Key Laboratory of ASIC and System, School of Microelectronics, Fudan University, Shanghai 200433, China; phzhang19@fudan.edu.cn

**Keywords:** MEMS accelerometers, sigma-delta, harmonic distortion, multiple degree-of-freedom model, finger flexibility

## Abstract

Harmonic distortion is one of the dominant factors limiting the overall signal-to-noise and distortion ratio of seismic-grade sigma-delta MEMS accelerometers. This study investigates harmonic distortion based on the multiple degree-of-freedom model (MDM) established in our previous study. The main advantage of using an MDM is that the effect of finger flexibility on harmonic distortion is considered. Initially, the nonlinear relationship between the input acceleration and output signal is derived using the MDM. Then, harmonic distortion is simulated and described in terms of the nonlinear input–output relationship. It is found that finger flexibility and parasitic capacitance mismatch both decrease harmonic distortion. Finally, the experimental testing of harmonic distortion is implemented. By reducing the finger length to realize a higher stiffness and compensating for the parasitic capacitance mismatch, the total harmonic distortion decreases from −66.8 dB to −86.9 dB.

## 1. Introduction

High-end capacitive accelerometers based on microelectromechanical system (MEMS) technology are widely applied in seismometers [1,2], inclination measurements [3], microgravity measurements [4], and inertial navigation [5], etc.

Low-noise MEMS accelerometers with a noise floor of sub-µg$/\sqrt{\mathrm{Hz}}$ are commonly required for seismic-grade applications [6]. As a result, the sensing element of seismic-grade MEMS accelerometers is usually vacuum-packaged to ensure very low Brownian noise [7,8]. A closed-loop control system is necessary for the vacuum-packaged sensing element to avoid unstable behavior, such as a long settling time and a significant overshoot [6]. The closed-loop solution based on the principle of electromechanical sigma-delta modulators has been widely used in MEMS capacitive accelerometers, which can provide a high-resolution digital output and possess advantages such as a high linearity and wide bandwidth [9].

For seismic-grade, high-precision sigma-delta accelerometers, noise and harmonic distortion are the dominant factors limiting the overall signal-to-noise and distortion ratio (SNDR) [8,10]. Many reports have been published on the mechanism and suppression of noise, including Brownian noise, quantization noise, and circuit noise [6]. Conversely, as the conversions between the sensing element and interface in the feedback loop and forward path are nonlinear, harmonic distortions appear in the output spectrum, which will decrease the SNDR [11]. Yu et al. first analyzed the nonlinear effects of the force feedback in a parallel-plate actuator and pick-off circuits, and a strategy for force feedback linearization was proposed and integrated into the process [10,12]. Xu et al. established a harmonic distortion model for sigma-delta accelerometers by considering the nonlinearity sources of the electrostatic feedback force and displacement-to-voltage conversion [11], and an interface circuit with force feedback linearization was also proposed [13]. Chen et al. studied the effect of parasitic capacitance mismatch on harmonic distortion and presented an online measuring and calibrating method for parasitic mismatch [14]. Chen et al. found a linear relationship between the second-order nonlinearity coefficient and the calibrating capacitance, and the calibrating process was optimized to reduce the second-order nonlinearity coefficient to the order of 10^−4^ [15].

Aside from force linearization based on an innovative circuit design, electrostatic force can also be linearized by the structural design of the sensing element. Amini et al. used a comb-drive actuator to avoid force nonlinearity [16]. The main advantage of a comb-drive actuator is that the electrostatic feedback force does not depend on the proof mass displacement, which provides linearity [16]. However, the electrostatic feedback force is lower than that of the parallel-plate actuator. Thus, branched comb-drive actuators using larger areas are usually employed to improve the electrostatic force [17,18,19,20,21,22].

Studies focusing on harmonic distortion are all based on the single degree-of-freedom model (SDM) of the sensing element. In our previous study [23], a multiple degree-of-freedom model (MDM), including finger flexibility, was established to analyze the noise. However, in this case, harmonic distortion was not investigated in detail. This study comprehensively investigates the mechanism and suppression of harmonic distortion using the MDM with parasitic mismatch. Compared to the SDM, the main advantage of the MDM is that the deterioration of harmonic distortion induced by finger flexibility can be included.

In Section 2, we describe how the nonlinear relationship between the input acceleration and output signal is derived using the MDM. Then, in Section 3, we describe how the harmonic distortion is simulated and its association with the nonlinear input–output relationship. Finally, the experimental testing of harmonic distortion is implemented to verify the theoretical results.

## 2. Nonlinear Relationship between the Input and Output

**Figure 1:**
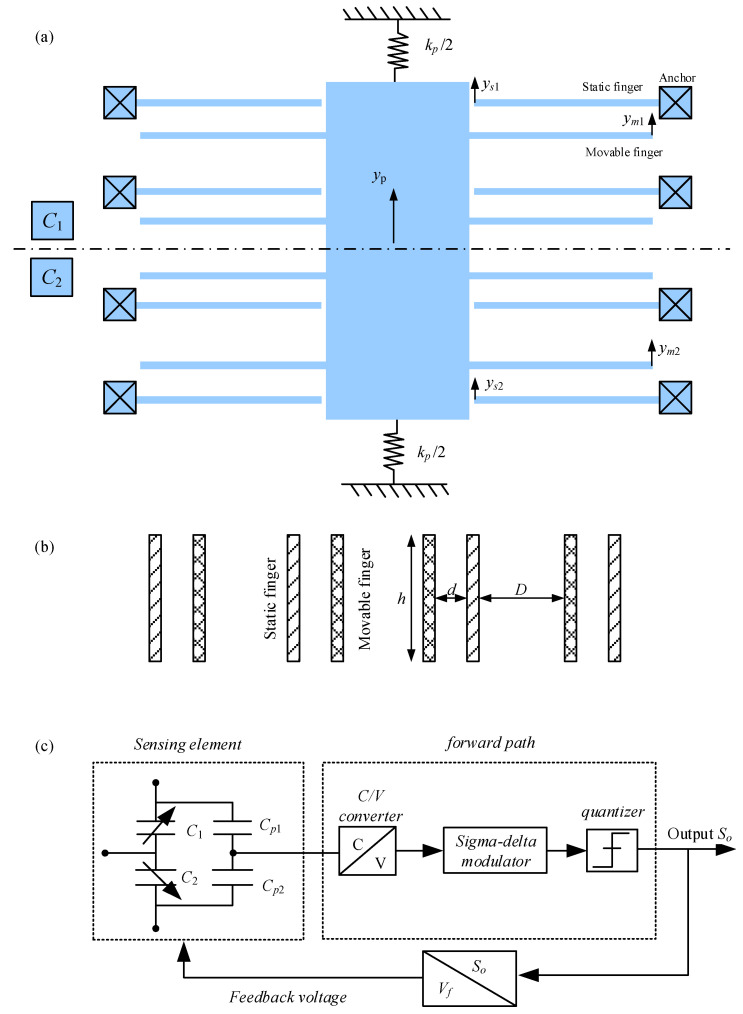
Structural diagram of the MEMS accelerometer. (**a**) Top view. (**b**) Cross-sectional view of fingers. (**c**) Differential detection considering the parasitic capacitance mismatch.

In this section, the nonlinear relationship between the input acceleration and output signal is derived based on the MDM established in our previous study [23]. By ignoring the acceleration and velocity terms of Equation (19) presented in [23], the steady-state equation of the sensing element is expressed as follows:

(1) $$\left[\begin{array}{ccccc} k_{p} & & & & \\ & k_{m} & & & \\ & & k_{m} & & \\ & & & k_{s} & \\ & & & & k_{s} \end{array}\right]\left[\begin{array}{c} y_{p}\\ y_{m1}\\ y_{m2}\\ y_{s1}\\ y_{s2} \end{array}\right]=\left[\begin{array}{c} Q_{ep}\\ Q_{m1}\\ Q_{m2}\\ Q_{s1}\\ Q_{s2} \end{array}\right]+\left[\begin{array}{c} F_{ep}\\ F_{em1}\\ F_{em2}\\ F_{es1}\\ F_{es2} \end{array}\right]$$

where *k_p_* denotes the stiffness of the spring supporting the proof mass; *k_m_* and *k_s_* denote the equivalent stiffness of movable and static fingers, respectively; *y_p_* indicates the displacement of the proof mass; *y_m_*_1_ and *y_m_*_2_ represent the displacements of the movable fingers belonging to *C*_1_ and *C*_2_, respectively; *y_s_*_1_ and *y_s_*_2_ denote the displacements of the static fingers belonging to *C*_1_ and *C*_2_, respectively, as shown in Figure 1; *Q_p_* denotes the inertial force applied to the proof mass induced by the input acceleration; *Q_m_*_1_ and *Q_m_*_2_ denote the inertial forces applied to the movable fingers belonging to *C*_1_ and *C*_2_, respectively; *Q_s_*_1_ and *Q_s_*_2_ denote the inertial forces applied to the static fingers belonging to *C*_1_ and *C*_2_, respectively; *F_ep_* denotes the electrostatic force applied to the proof mass; *F_em_*_1_ and *F_em_*_2_ denote the electrostatic forces applied to the movable fingers belonging to *C*_1_ and *C*_2_, respectively; and *F_es_*_1_ and *F_es_*_2_ denote the electrostatic forces applied to the static fingers belonging to *C*_1_ and *C*_2_, respectively.

The expressions of *Q_p_* and the electrostatic forces givenin the previous study [23] are rewritten here

(2) $$Q_{p}=-m_{t}a$$

(3) $$\begin{array}{l} F_{ep}=F_{e0}{\left(S_{o}-1\right)}^{2}\left[1/{\left(1-{\tilde{y}}_{p}\right)}^{2}-1/{\left(D/d+{\tilde{y}}_{p}\right)}^{2}+2\left(r_{m}{\tilde{y}}_{m1}-r_{s}{\tilde{y}}_{s1}\right)/{\left(1-{\tilde{y}}_{p}\right)}^{3}\right]\\ -F_{e0}{\left(S_{o}+1\right)}^{2}\left[1/{\left(1+{\tilde{y}}_{p}\right)}^{2}-1/{\left(D/d-{\tilde{y}}_{p}\right)}^{2}-2\left(r_{m}{\tilde{y}}_{m2}-r_{s}{\tilde{y}}_{s2}\right)/{\left(1+{\tilde{y}}_{p}\right)}^{3}\right] \end{array}$$

(4) $$F_{em1}=F_{e0}{\left(S_{o}-1\right)}^{2}\left[r_{m}/{\left(1-{\tilde{y}}_{p}\right)}^{2}-r_{m}/{\left(D/d+{\tilde{y}}_{p}\right)}^{2}+2\left(r_{mm}{\tilde{y}}_{m1}-r_{ms}{\tilde{y}}_{s1}\right)/{\left(1-{\tilde{y}}_{p}\right)}^{3}\right]$$

(5) $$F_{em2}=-F_{e0}{\left(S_{o}+1\right)}^{2}\left[r_{m}/{\left(1+{\tilde{y}}_{p}\right)}^{2}-r_{m}/{\left(D/d-{\tilde{y}}_{p}\right)}^{2}-2\left(r_{mm}{\tilde{y}}_{m2}-r_{ms}{\tilde{y}}_{s2}\right)/{\left(1+{\tilde{y}}_{p}\right)}^{3}\right]$$

(6) $$F_{es1}=-F_{e0}{\left(S_{o}-1\right)}^{2}\left[r_{s}/{\left(1-{\tilde{y}}_{p}\right)}^{2}-r_{s}/{\left(D/d+{\tilde{y}}_{p}\right)}^{2}+2\left(r_{sm}{\tilde{y}}_{m1}-r_{ss}{\tilde{y}}_{s1}\right)/{\left(1-{\tilde{y}}_{p}\right)}^{3}\right]$$

(7) $$F_{es2}=F_{e0}{\left(S_{o}+1\right)}^{2}\left[r_{s}/{\left(1+{\tilde{y}}_{p}\right)}^{2}-r_{s}/{\left(D/d-{\tilde{y}}_{p}\right)}^{2}-2\left(r_{sm}{\tilde{y}}_{m2}-r_{ss}{\tilde{y}}_{s2}\right)/{\left(1+{\tilde{y}}_{p}\right)}^{3}\right]$$

where *m_t_* denotes the mass sum of the proof mass and all the movable fingers; *a* represents the input acceleration; *F*_e0_ denotes the rest electrostatic force; *S_o_* denotes the output; *d* and *D* denote the narrow and wide gaps, respectively, as shown in Figure 1; and *r_m_*, *r_s_*, *r_mm_*, *r_ms_*, *r_sm_*, and *r_ss_* denote the length coefficients. The normalized displacements ${\tilde{y}}_{p},{\tilde{y}}_{m1},{\tilde{y}}_{m2},{\tilde{y}}_{s1},{\tilde{y}}_{s2}$ are expressed as follows:

(8) $${\tilde{y}}_{p}=y_{p}/d,{\tilde{y}}_{m1}=y_{m1}/d,{\tilde{y}}_{m2}=y_{m2}/d,{\tilde{y}}_{s1}=y_{s1}/d,{\tilde{y}}_{s2}=y_{s2}/d$$

Detailed expressions of the other parameters, including the stiffness, inertial forces, and rest electrostatic force, etc., are all presented in the previous study [23].

Because the normalized displacements ${\tilde{y}}_{p},{\tilde{y}}_{m1},{\tilde{y}}_{m2},{\tilde{y}}_{s1},{\tilde{y}}_{s2}$re much smaller than 1, the dependence of the electrostatic forces on the normalized displacements can be ignored. For the expressions of the electrostatic forces Equations (3)–(7), the portions resulting from the wide gap, which are inversely proportional to the square of *D/d*, can also be ignored, because the gap ratio *D/d* is much greater than 1. As a whole, the expressions of the electrostatic forces can be simplified into:

(9) $$\begin{array}{l} F_{ep}=F_{e0}{\left(S_{o}-1\right)}^{2}\left[1/{\left(1-{\tilde{y}}_{p}\right)}^{2}+2\left(r_{m}{\tilde{y}}_{m1}-r_{s}{\tilde{y}}_{s1}\right)/{\left(1-{\tilde{y}}_{p}\right)}^{3}\right]\\ -F_{e0}{\left(S_{o}+1\right)}^{2}\left[1/{\left(1+{\tilde{y}}_{p}\right)}^{2}-2\left(r_{m}{\tilde{y}}_{m2}-r_{s}{\tilde{y}}_{s2}\right)/{\left(1+{\tilde{y}}_{p}\right)}^{3}\right] \end{array}$$

(10) $$F_{em1}=F_{e0}{\left(S_{o}-1\right)}^{2}r_{m}/{\left(1-{\tilde{y}}_{p}\right)}^{2}$$

(11) $$F_{em2}=-F_{e0}{\left(S_{o}+1\right)}^{2}r_{m}/{\left(1+{\tilde{y}}_{p}\right)}^{2}$$

(12) $$F_{es1}=-F_{e0}{\left(S_{o}-1\right)}^{2}r_{s}/{\left(1-{\tilde{y}}_{p}\right)}^{2}$$

(13) $$F_{es2}=F_{e0}{\left(S_{o}+1\right)}^{2}r_{s}/{\left(1+{\tilde{y}}_{p}\right)}^{2}$$

Substituting Equations (2) and (9) into (1) and eliminating the finger displacements *y_m_*_1_, *y_m_*_2_, *y_s_*_1_, and *y_s_*_2_ leads to the following:

(14) $$\begin{array}{l} k_{p}y_{p}=-m_{t}a+F_{e0}{\left(S_{o}-1\right)}^{2}\left[1/{\left(1-{\tilde{y}}_{p}\right)}^{2}+2\left(\frac{r_{m}}{dk_{m}}\left(Q_{m1}+F_{em1}\right)-\frac{r_{s}}{dk_{s}}\left(Q_{s1}+F_{es1}\right)\right)/{\left(1-{\tilde{y}}_{p}\right)}^{3}\right]\\ -F_{e0}{\left(S_{o}+1\right)}^{2}\left[1/{\left(1+{\tilde{y}}_{p}\right)}^{2}-2\left(\frac{r_{m}}{dk_{m}}\left(Q_{m2}+F_{em2}\right)-\frac{r_{s}}{dk_{s}}\left(Q_{s2}+F_{es2}\right)\right)/{\left(1+{\tilde{y}}_{p}\right)}^{3}\right] \end{array}$$

According to the results from the previous study [23], the following equations are valid:

(15) $$Q_{m1}=Q_{s1}=Q_{m2}=Q_{s2},r_{m}=r_{s},k_{m}=k_{s}$$

Substituting Equations (10)–(13) and (15) into (14) leads to the following:

(16) $$m_{t}a=-k_{p}d{\tilde{y}}_{p}+F_{e0}{\left(S_{o}-1\right)}^{2}\left[\frac{1}{{\left(1-{\tilde{y}}_{p}\right)}^{2}}+\beta \frac{{\left(S_{o}-1\right)}^{2}}{{\left(1-{\tilde{y}}_{p}\right)}^{5}}\right]-F_{e0}{\left(S_{o}+1\right)}^{2}\left[\frac{1}{{\left(1+{\tilde{y}}_{p}\right)}^{2}}+\beta \frac{{\left(S_{o}+1\right)}^{2}}{{\left(1+{\tilde{y}}_{p}\right)}^{5}}\right]$$

where *β* represents the impact of the finger flexibility regarding the nonlinear relationship and is inversely proportional to the finger stiffness *k_m_*.

(17) $$\beta =\frac{4r_{m}^{2}F_{e0}}{dk_{m}}$$

According to the detecting principle of accelerometers, the differential capacitance of the sensing element is expressed as follows:

(18) $$\Delta C=\frac{2N\varepsilon hl}{d}{\tilde{y}}_{p}+\Delta C_{p}$$

where the tiny dependences of the differential difference on the normalized finger displacements and wide gaps are also ignored, similar to the simplification of the electrostatic forces, and Δ*C_p_* represents the parasitic capacitance mismatch. Based on the differential difference, the output signal *S_o_* is expressed as follows:

(19) $$S_{o}=G\Delta C$$

where *G* denotes the low-frequency gain of the forward path transfer function, as shown in Figure 1c. Substituting Equation (18) into (19) leads to the following:

(20) $${\tilde{y}}_{p}=\frac{S_{o}}{2G\left(N\varepsilon hl/d\right)}-\frac{\Delta C_{p}}{2\left(N\varepsilon hl/d\right)}=\frac{S_{o}}{H}-\frac{\Delta C_{p}}{2C_{0}}$$

where *H* denotes the gain from the normalized displacement to the output and *C_0_* denotes the rest capacitance.

Substituting Equation (20) into (16) and then using Taylor’s expansion law leads to the following:

(21) $$\begin{array}{cc} & m_{t}a=k_{0}+k_{1}S_{o}+k_{2}S_{o}^{2}+k_{3}\frac{S_{o}^{3}}{H}+k_{4}\frac{S_{o}^{4}}{H^{2}}+k_{5}\frac{S_{o}^{5}}{H^{3}}+k_{6}\frac{S_{o}^{6}}{H^{4}}+k_{7}\frac{S_{o}^{7}}{H^{5}}\\ & where,\;k_{0}=\left(k_{p}d-4F_{e0}-10\beta F_{e0}\right)\frac{\Delta C_{p}}{2C_{0}},\;k_{1}=-\left(4+8\beta \right)F_{e0}\\ & k_{2}=-\left(4+60\beta \right)F_{e0}\frac{\Delta C_{p}}{2C_{0}},\;k_{3}=\left(4+8H\beta \right)F_{e0}\\ & k_{4}=-\left(24+10H^{2}\beta \right)F_{e0}\frac{\Delta C_{p}}{2C_{0}},\;k_{5}=\left(8+10H^{2}\beta \right)F_{e0}\\ & k_{6}=-\left(60+210H^{2}\beta \right)F_{e0}\frac{\Delta C_{p}}{2C_{0}},\;k_{7}=\left(12+70H^{2}\beta \right)F_{e0} \end{array}$$

Firstly, it can be seen in Equation (21) that *β* increases the nonlinear terms’ coefficients. Because the coefficient *β* is inversely proportional to the finger stiffness *k_m_*, as shown in Equation (17), the finger flexibility enhances the nonlinearity of the input–output relationship. Additionally, compared to the quadratic and cubic terms, the nonlinear terms with a higher degree than three are more susceptible to finger flexibility because the gain *H* is much higher than 1. Secondly, it can also be seen that the parasitic capacitance mismatch Δ*C_p_* induces even nonlinear terms.

## 3. Harmonic Distortion Simulation and Discussion

In this section, the harmonic distortion of MEMS accelerometers is simulated, and the simulated results are discussed with reference to the nonlinear input–output relationship.

**Figure 2:**
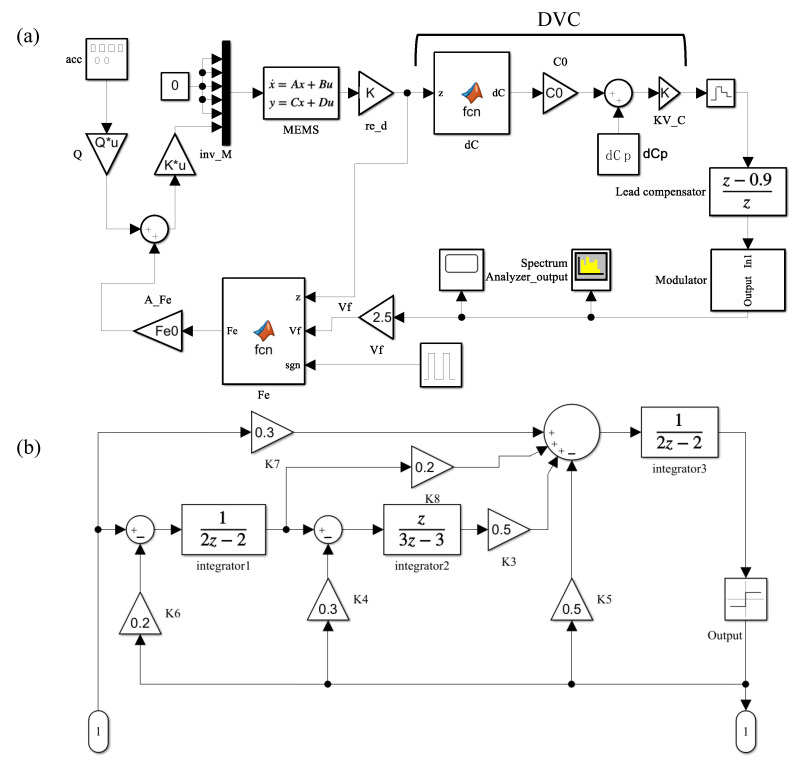
The system that uses the MDM of the sensing element. (**a**) Overall system and (**b**) sigma–delta modulator. DVC denotes the displacement–voltage converter block and dCp denotes the parasitic capacitance mismatch.

Based on the MDM, the sigma-delta system for simulating the harmonic distortion of accelerometers is established using Simulink, as shown in Figure 2. As in the previous study [23], the system consists of an MEMS sensing element, a displacement–voltage converter, a zero-order holder, a lead compensator, a third-order sigma-delta modulator, and an electrostatic force block. The only difference is that the simulating system for the harmonic distortion system contains parasitic capacitance mismatch. The 1-bit bitstream output of the sigma-delta modulator is adopted as the overall system’s output.

The parameters adopted in the simulation are listed in Table 1. The input acceleration has an amplitude of 0.5 g and a frequency of 50 Hz. The sampling frequency *f_s_* is 250 kHz. The finger length *l* and parasitic capacitance mismatch Δ*C_p_* are set as variables to check their effects on the harmonic distortion. Decreasing the length can notably improve the finger stiffness *k_m_* because the stiffness is inversely proportional to the square of the length [23]. In this study, two versions of accelerometers with finger lengths of 325 μm and 175 μm are designed. However, the shorter finger results in a lower sensitivity because the capacitance is directly proportional to the finger length. When the finger length decreases from 325 μm to 175 μm, the number of fingers increases from 288 to 544 to maintain the sensitivity at a constant level.

**Figure 3:**
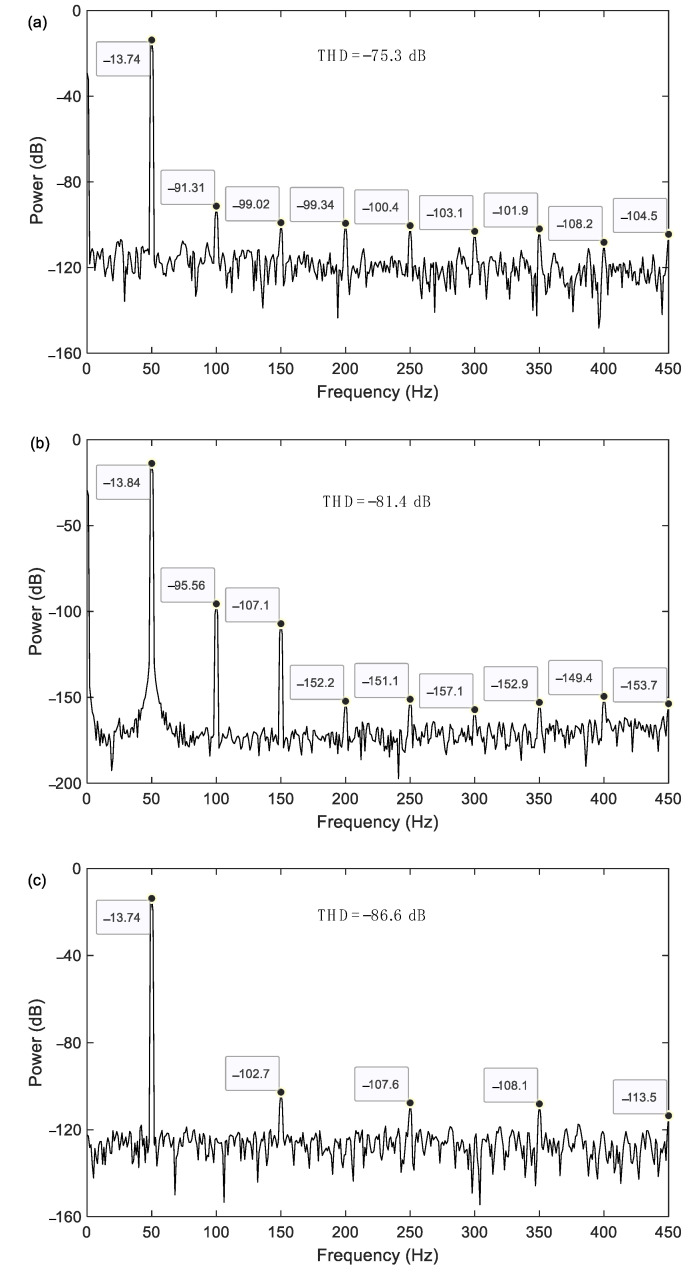
Simulated power spectrum of the 1-bit bitstream output; (**a**) finger length *l* = 325 μm, number of movable fingers *N* = 288, and parasitic capacitance mismatch Δ*C_p_* = 0.1 pF; (**b**) finger length *l* = 175 μm, number of movable fingers *N* = 544, and parasitic capacitance mismatch Δ*C_p_* = 0.1 pF; and (**c**) finger length *l* = 325 μm, number of movable fingers *N* = 288, and parasitic capacitance mismatch Δ*C_p_* = 0.0 pF.

After the simulation using the parameters listed in Table 1, the simulated power spectrum of the 1-bit bitstream output is shown in Figure 3. It can be seen that the output signal contains a fundamental response of 50 Hz and harmonics of 100 Hz, 150 Hz, 200 Hz, 250 Hz, and 300 Hz, etc. These harmonics are undoubtedly the source of the harmonic distortion. The total harmonic distortion (THD) can be expressed as follows:

(22) $$THD=20\mathrm{lg}\left(\sqrt{\frac{\sum_{i=2}^{n}{\left(10^{V_{i}/20}\right)}^{2}}{{\left(10^{V_{1}/20}\right)}^{2}}}\right)\left(\mathrm{dB}\right)$$

where *V*_1_ denotes the fundamental response and *V_i_* represents the *i*th*-*degree harmonic.

In Figure 3a,b, when the finger length decreases from 325 μm to 175 μm to improve the stiffness, the harmonics significantly decline, especially those with a degree higher than three. The THD also decreases from −75.3 dB to −81.4 dB. The mechanism of this is that the finger flexibility enhances the nonlinearity of the input–output relationship, especially for the nonlinear terms with a degree higher than three. In other words, a higher stiffness can undoubtedly suppress the increase in nonlinearity. Furthermore, from Figure 3a,c, it can be seen that the parasitic capacitance mismatch induces notable even harmonics, and the THD also decreases from −75.3 dB to −86.6 dB after the parasitic capacitance mismatch decreases from 0.1 pF to zero. The reason for this is that the parasitic capacitance mismatch is the cause of even nonlinear terms of the input–output relationship.

As a whole, finger flexibility and parasitic capacitance mismatch both have significant effects on harmonic distortion. Therefore, improving the finger stiffness and compensating for the parasitic capacitance mismatch are both necessary to suppress harmonic distortion.

## 4. Experimental Testing

**Figure 4:**
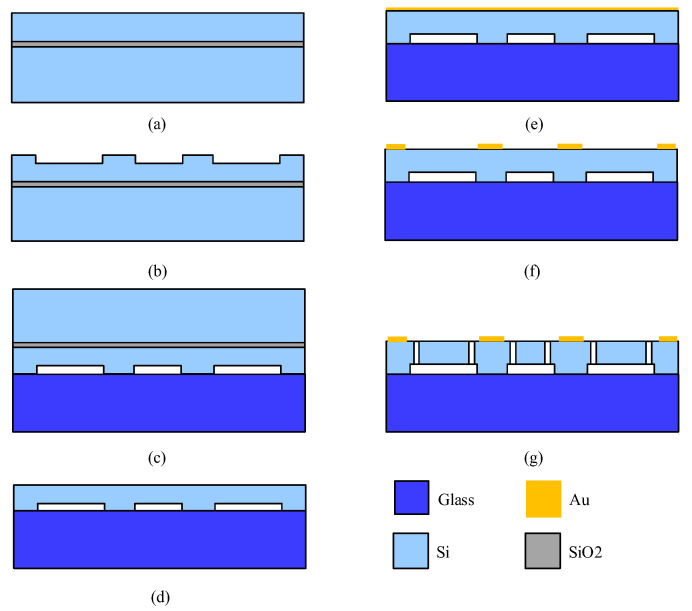
The fabrication process for the sensing element.

The sensing element of the MEMS accelerometers was fabricated using a silicon-on-glass process, as shown in Figure 4. Compared to the silicon-on-insulator (SOI) process requiring releasing holes in the device layer [24], the silicon-on-glass process does not etch releasing holes in the device layer, and consequently produces a more compact device. The fabrication process started with an SOI wafer (Figure 4a). Firstly, deep reactive ion etching was employed to fabricate an anchor with a height of 20 μm (Figure 4b). Secondly, the SOI wafer was flipped and bonded on a borosilicate glass wafer using anodic bonding (Figure 4c), and the substrate and sacrificial layers of the SOI wafer were removed via wet etching (Figure 4d). Thirdly, a metal layer was sputtered on the structural layer (Figure 4e), and wet etching was used to strip the metal layer to pattern the pads for interconnection (Figure 4f). Finally, the structural layer was etched and released using deep reactive ion etching (Figure 4g).

**Figure 5:**
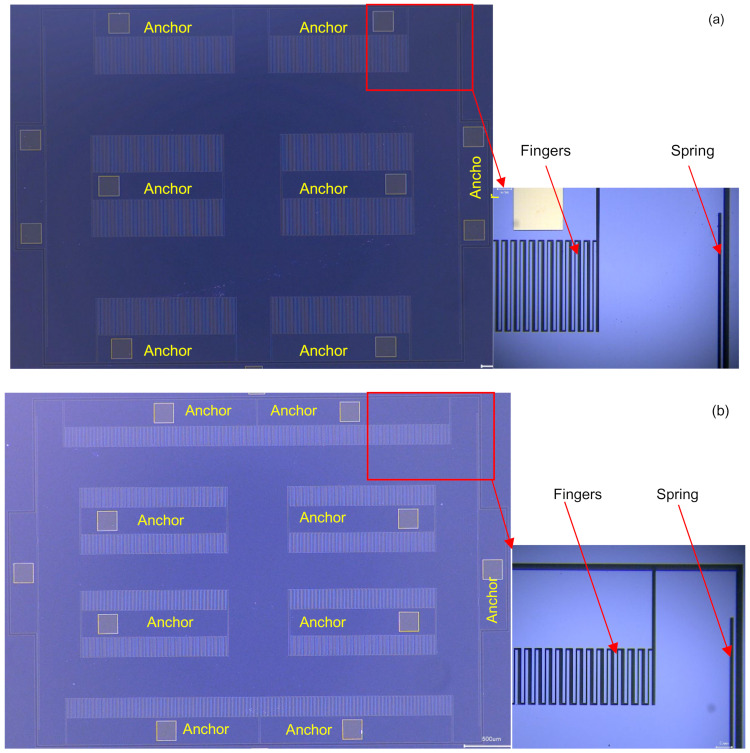
Microscope pictures of the sensing elements, (**a**) long-finger version with a finger length of 325 μm and (**b**) short-finger version with a finger length of 175 μm.

The microscope pictures of the sensing elements are shown in Figure 5. Two versions of the sensing element with different finger lengths were fabricated in this study. The finger length of the long-finger version was 325 μm, and that of the short-finger version was 175 μm. To ensure the two versions had the same capacitance, the long- and short-finger versions had 288 and 544 movable fingers, respectively. The MEMS sensing element of the accelerometers was encapsulated using ceramic vacuum packaging. First, the sensing element was fixed on the substrate of the ceramic package using an adhesive, and the package was then placed into a thermal chamber to solidify the adhesive. Then, wire bonding was performed to interconnect the package and the sensing element. Finally, vacuum pumping and cap sealing were performed to encapsulate the package. The two versions of the sensing elements were packaged with the same vacuum level to make them both have high Q.

**Figure 6:**
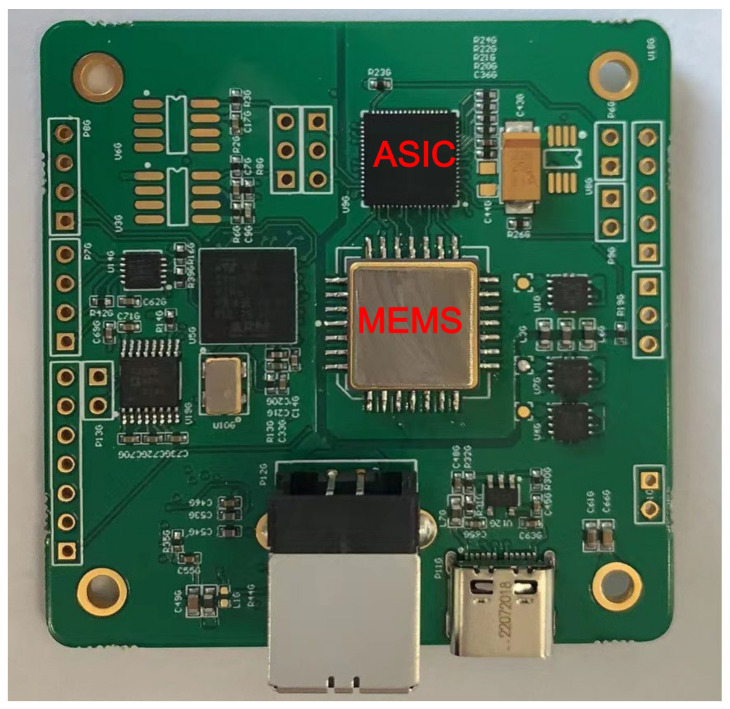
Printed circuit board with MEMS sensing element and AISC chip of the interface circuit.

In this study, a closed-loop interface circuit, developed by Prof. Yin and Fu from the MEMS center of the Harbin Institute of Technology of China, was adopted to configure the accelerometers [11,14]. The interface circuit realizes a five-order sigma-delta scheme to shape the noise. Additionally, the interface circuit can calibrate the parasitic capacitance mismatch to suppress even-degree harmonics based on the harmonic distortion self-test. The AISC chip of the interface circuit and packaged MEMS sensing element were assembled using a printed circuit board, as shown in Figure 6.

To study the effect of the parasitic mismatch on the harmonic distortion, the parasitic capacitance mismatch of the short-finger version was calibrated to suppress the even-degree harmonics, but the long-finger version was not. A 200 mV exciting signal was applied at 100 Hz to the self-test circuit to perform an input acceleration to test the distortion. The sampling frequency of the interface circuit was 250 kHz, and an Agilent Logic Analyzer sampled the 1-bit digital output bitstream of the accelerometers. Then, the sampled data were input into the commercial software MATLAB R2019a, where the spectrum-analyzing function was used to evaluate the power spectrum.

**Figure 7:**
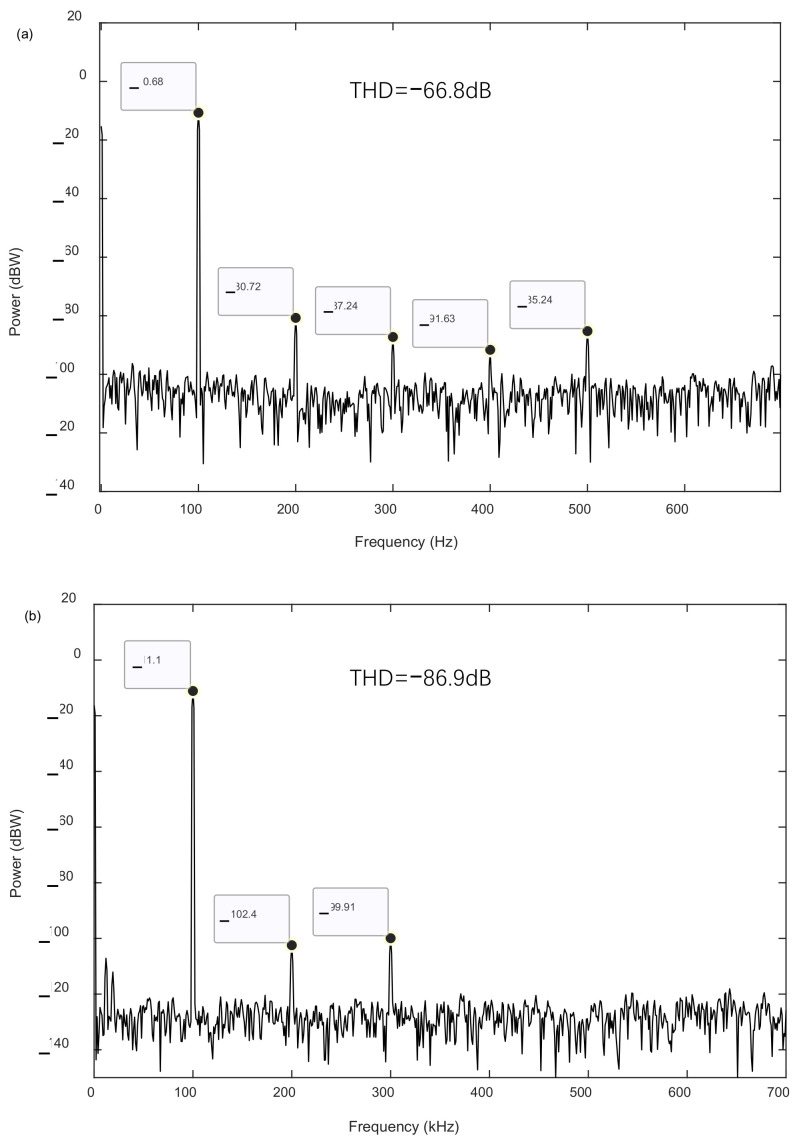
The testing results of the harmonics of accelerometers; (**a**) long-finger version with a finger length of 325 μm and (**b**) short-finger version with a finger length of 175 μm.

The obtained results of the power spectrum for the two versions of the accelerometers are shown in Figure 7. Compared to the long-finger version, the even-degree harmonics of the short-finger version accelerometer were lower. For instance, the quadratic harmonics of the short-finger and long-finger versions were −102.4 dB and −80.7 dB, respectively. Thus, the testing result of the even-degree harmonics verified that the parasitic capacitance mismatch induced significant even-degree harmonic distortion, which coincides with the theoretical results described in Section 4.

Furthermore, compared to the long-finger version, the odd-degree harmonics of the short-finger version accelerometer were also significantly lower, especially for the harmonics with a higher degree than three. This testing result verified that the finger flexibility deteriorated the harmonic distortion of the accelerometers, and that improving the finger stiffness could suppress the harmonic distortion.

**Figure 8:**
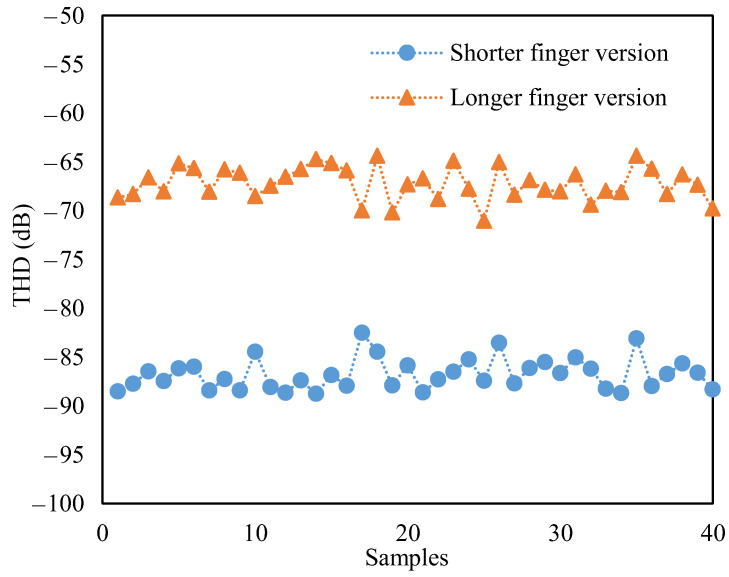
THDs obtained from additional testing of harmonic distortion.

Finally, to compare the THDs of the two versions of accelerometers, the amplitudes of the fundamental response and harmonics were substituted into Equation (22) to evaluate the THD, and the estimated results are also listed in Figure 7. Compared to the long-finger version with a THD of −66.8 dB, the THD of the short-finger version had a much lower THD of −86.9 dB. Thus, the decrease in the THD approached 20.1 dB. The measuring uncertainties, such as the environmental vibration and signal noise, may induce errors on the THD. To evaluate the effect of the measuring uncertainties, 40 additional times of harmonic distortion testing were carried out, and the obtained THDs are shown in Figure 8. Using the formula for the confidence interval [25] and confidence level of 90%, the computational confidence intervals of the longer-finger and shorter-finger versions were [−67.62 dB, −66.74 dB] and [−87.19 dB, −86.35 dB], respectively. Additionally, the THD standard deviations of the longer-finger and shorter-finger versions were 1.70 dB and 1.61 dB, respectively. The standard deviations induced by the measuring uncertainties were much smaller than the THD decrease. Thus, the technique of suppressing harmonic distortion was reliable.

Overall, the finger flexibility and parasitic capacitance mismatch decreased the harmonic distortion. Therefore, improving the finger stiffness and calibrating the parasitic mismatch could suppress the harmonic distortion.

## 5. Conclusions

As one of the dominant factors limiting the SNDR, the harmonic distortion of seismic-grade sigma-delta accelerometers was comprehensively investigated using the MDM. It was found that finger flexibility enhances the harmonics of an output signal, especially those with a degree higher than three. The mechanism is that the finger flexibility enhances the nonlinearity of the input–output relationship, especially for nonlinear terms with a degree higher than three. Additionally, the parasitic capacitance mismatch induces notable even harmonics. This is because the parasitic capacitance mismatch is the cause of even nonlinear terms of the input–output relationship. As a result, improving the finger stiffness and compensating for the parasitic capacitance mismatch are both necessary to suppress harmonic distortion. The harmonic distortion was tested to verify the theoretical results. It was shown that the THD decreased from −66.8 dB to −86.9 dB after improving the finger stiffness and compensating for the parasitic capacitance mismatch.

In the future, optimizing accelerometers using the MDM to ensure a lower harmonic distortion is a valuable option. Investigating the harmonic distortion of sigma–delta gyroscopes using the MDM is also beneficial.

## Figures and Tables

**Table 1:** Parameters for simulation.

Parameters	Value	Unit
Density (*ρ*)	2330	kg/m^3^
Young’s modulus (*E*)	169	GPa
Length of spring (*L_s_*)	885	μm
Width of spring (*w_s_*)	9	μm
Proof mass (*m_p_*)	9.11 × 10^−7^	kg
Width of movable and static fingers (*w*)	10	μm
Height of fingers (*h*)	60	μm
Narrow gap (*d*)	3	μm
Wide gap (*D*)	10	μm
Feedback voltage (*V_f_*)	2.5	V
The dielectric constant of air (ε)	8.854 × 10^−12^	F/m
Sampling frequency (*f_s_*)	250	kHz
Capacitance–voltage conversion	6.67	V/pf
Quality factor (*Q*)	2000	--

## Data Availability

Data would be available upon request on a personal contact with the corresponding author at the email address: xuefengwang19@fudan.edu.cn.
